# Using Large Language Models to Retrieve Critical Data from Clinical Processes and Business Rules

**DOI:** 10.3390/bioengineering12010017

**Published:** 2024-12-28

**Authors:** Yunguo Yu, Cesar A. Gomez-Cabello, Svetlana Makarova, Yogesh Parte, Sahar Borna, Syed Ali Haider, Ariana Genovese, Srinivasagam Prabha, Antonio J. Forte

**Affiliations:** 1 Center for Digital Health, Mayo Clinic, Rochester, MN 55905, USA; 2 Division of Plastic Surgery, Mayo Clinic, 4500 San Pablo Road, Jacksonville, FL 32224, USA

**Keywords:** diagnostics, clinical decision support, Artificial Intelligence, large language models, data retrieval

## Abstract

Current clinical care relies heavily on complex, rule-based systems for tasks like diagnosis and treatment. However, these systems can be cumbersome and require constant updates. This study explores the potential of the large language model (LLM), LLaMA 2, to address these limitations. We tested LLaMA 2′s performance in interpreting complex clinical process models, such as Mayo Clinic Care Pathway Models (CPMs), and providing accurate clinical recommendations. LLM was trained on encoded pathways versions using DOT language, embedding them with SentenceTransformer, and then presented with hypothetical patient cases. We compared the token-level accuracy between LLM output and the ground truth by measuring both node and edge accuracy. LLaMA 2 accurately retrieved the diagnosis, suggested further evaluation, and delivered appropriate management steps, all based on the pathways. The average node accuracy across the different pathways was 0.91 (SD ± 0.045), while the average edge accuracy was 0.92 (SD ± 0.122). This study highlights the potential of LLMs for healthcare information retrieval, especially when relevant data are provided. Future research should focus on improving these models’ interpretability and their integration into existing clinical workflows.

## 1. Introduction

In the healthcare sector, the design and execution of clinical processes are predominantly governed by rule-based systems [1]. These systems, which encapsulate the subtleties of medical decision-making, are pivotal in various facets of patient care, including disease diagnosis and treatment [1,2,3]. For instance, consider the diagnostic process for coronavirus disease 2019 (COVID-19), a disease that has profoundly impacted global health systems [4,5]. This process typically involves a complex set of rules that take into account a patient’s current symptoms, vaccination history (including COVID-19 vaccinations), the severity of their condition, and the need to differentiate COVID-19 from other respiratory illnesses such as influenza or non-COVID-19 lung infections [5,6]. These rules are meticulously crafted based on clinical guidelines, best practices, and expert knowledge to ensure patients receive timely and accurate care [7,8].

Despite the effectiveness of rule-based systems in guiding clinical decision-making, they are not without limitations [8,9]. One of the primary challenges is the overwhelming volume and complexity of rules required to cover the wide array of clinical scenarios encountered in practice [7]. As our understanding of diseases and treatments evolves, maintaining and updating these rules becomes increasingly challenging. Additionally, rule-based systems may struggle to capture the nuances and context-dependent nature of medical decision-making, leading to suboptimal outcomes in certain situations [10].

The emergence of large language models (LLMs) has demonstrated remarkable logical reasoning capabilities, even for complex clinical rules [11,12]. LLMs have shown their potential as pivotal tools in medical practice by excelling in the distinct nuances of clinical decision support, even without the implementation of fine-tuning techniques [12,13,14,15,16,17,18]. Related studies show that LLMs can supplement traditional Clinical Decision Support Systems’ (CDSSs) optimization by decreasing the number of unnecessary alerts and providing relevant, understandable, and non-redundant recommendations compared to human systems [19]. Furthermore, they can accurately provide timely and individualized diagnosis and treatment [11,20,21]. However, there were some gaps and deficiencies in their knowledge, highlighting their lack of training for these tasks. Conversely, techniques to improve LLMs’ understanding and performance, such as fine-tuning and Retrieval-Augmented Generation (RAG), enable them to better understand the context, input, and output in a specific domain [22,23,24,25,26].

**Figure 1:**
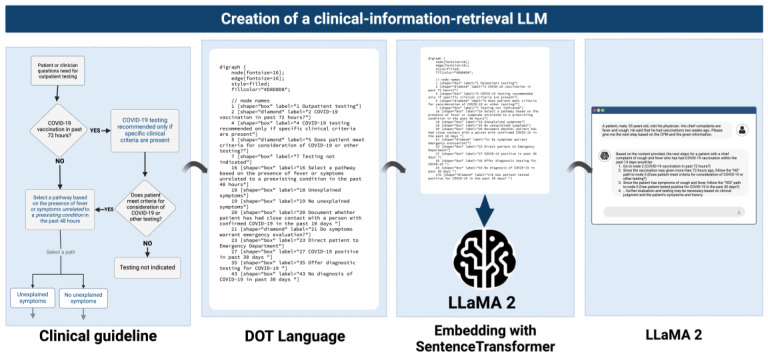
A summary of the methodology for creating a large language model to retrieve clinical information. First, we select clinical guidelines based on the Mayo Clinic consensus. Second, the guidelines are coded in DOT language (see Supplementary Material). Third, the encoded guidelines are embedded using HuggingFace SentenceTransformer. Finally, the large language model interprets the encoded processes and identifies critical steps for diagnosis and management. Created in BioRender [27].

This paper showcases how LLMs can be leveraged to retrieve information from complex rule-based systems in healthcare thereby enhancing information systems. The process involves encoding complex clinical rules, such as those in Care Pathway Models (CPMs), into DOT (graph description language) language, followed by embedding the encoded rules into a vector database. Subsequently, an LLM—LLaMA 2—is employed to retrieve the information to support clinical decision-making (Figure 1). Finally, we analyze the performance of the model by measuring its accuracy at a token-level. By harnessing the power of LLMs, healthcare providers can potentially enhance the retrieval of critical information from clinical processes and business rules, leading to more efficient and effective decision-making. This paper explores the potential of LLMs to retrieve critical information from complex clinical processes and business rules for improving clinical decision support and reducing physicians’ administrative burdens.

## 2. Methods

### 2.1. Selection of LLM

The large language model (LLM) selected for this study is the open-source, publicly available LLAMA 2 model. LLAMA 2 wass chosen for its robust performance in natural language understanding and generation tasks, making it suitable for retrieving information from complex clinical processes and business rules.

### 2.2. Dataset

The clinical guidelines and recommendations used in this study are based on the Mayo Clinic consensus [28], which was derived from a review of existing evidence and guidelines. It is important to note that these recommendations do not replace clinical judgment but serve as a guideline for healthcare professionals. While several CPMs were evaluated to demonstrate the application of the model, we demonstrate a clinical scenario related to the diagnosis and testing of COVID-19 in adults, specifically outpatient testing.

### 2.3. Encoding the Clinical Procedures

**Figure 2:**
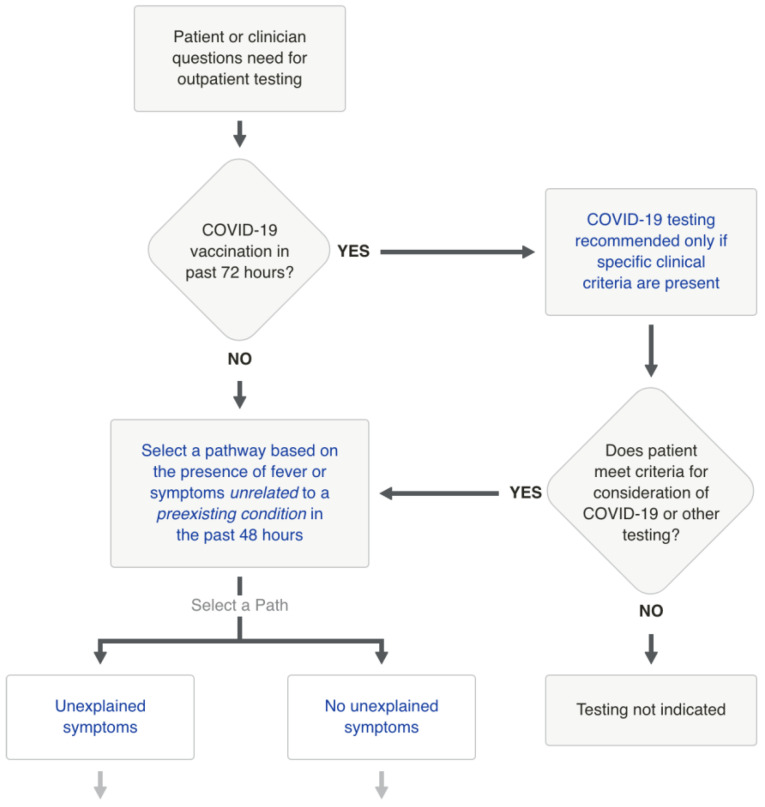
Example of partial Care Pathway Model (CPM) for COVID-19 testing for adults. From original algorithm obtained from AskMayoExpert.

To encode the clinical recommendations provided by CPMs, we utilize DOT language, a format commonly used to represent graphical models, including rules and decision trees. In DOT language, there are three kinds of objects: graphs, nodes, and edges. A node is created when it first appears in the file and an edge is created when nodes are joined by the “->” operator [29]. In the scenario depicted in Figure 2, examples of nodes are the question regarding the presence of COVID-19 vaccination, the presence of symptoms in the last 48 h, and the recommendation of testing with specific clinical criteria. Conversely, edges are the relationship between those three questions.

The clinical procedures were sourced from the AskMayoExpert site [30], which provides detailed clinical guidelines and recommendations for healthcare professionals (Figure 2). See Supplementary Material for a sample of a CPM (COVID-19 testing for adults) in DOT language.

### 2.4. Embedding the Encoded Processes

The encoded clinical processes are then embedded using HuggingFace SentenceTransformer, a library designed for encoding sentences and paragraphs into fixed-dimensional embeddings. This step is crucial for transforming the textual representation of clinical procedures into a format that the LLM can process.

### 2.5. Information Retrieval Approach

The methodology for using LLM to retrieve critical steps from the encoded clinical processes involves the following steps (Figure 1): First, we input the encoded clinical processes into the LLAMA 2 model. Once in the model, we leveraged the LLM’s natural language understanding capabilities to interpret the encoded processes and identify critical steps. Finally, we can retrieve the critical steps based on the given contents, focusing on key aspects such as patient symptoms, vaccination history, severity of condition, and differentiation from other respiratory illnesses.

**Figure 3:**
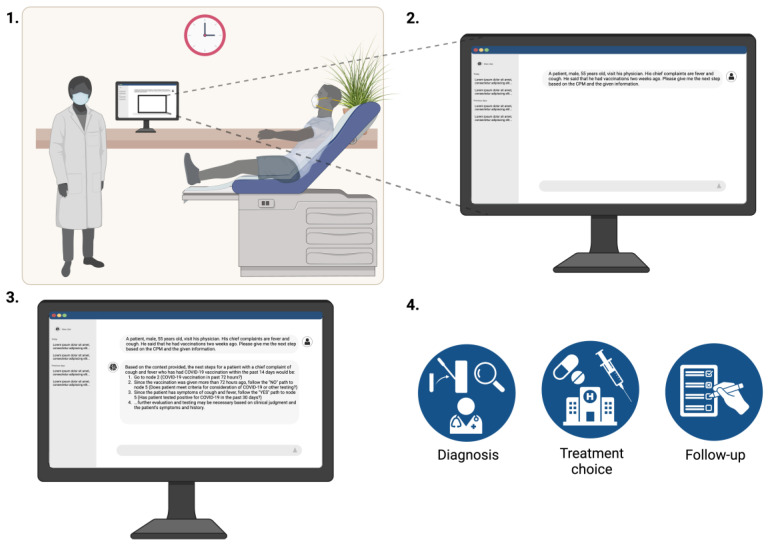
The use of large language models in clinical practice. The patient comes to the clinic or emergency room and is evaluated by the clinician (1), who concurrently inputs the clinical presentation to the large language model-based clinical decision system (2). The system provides individualized recommendations for diagnosis and treatment based on its specialized training data (3), which will ultimately help the physician determine the appropriate management (4). Created in BioRender [27].

By following this approach, we aim to demonstrate the effectiveness of using LLMs for retrieving critical information from complex clinical processes and business rules (Figure 3).

### 2.6. Measuring Model Accuracy

We compared the token-level accuracy between LLM output and the ground truth by measuring both node and edge accuracy. Ground truth was provided by the DOT language files obtained from the CPMs. Nodes are identified as individual entities, and edges are connections or relationships that link pairs of nodes. First, we parsed the DOT and LLM-generated JSON (JavaScript Object Notation) files describing the graph structures using nodes and edges. Then, we compared the nodes from the DOT files with those from the JSON outputs to determine the overlap. This is performed by calculating the intersection of node sets from both sources. Similarly, edges were compared between the two files, with the intersection of edge sets used to identify matches. Accuracy is computed as the ratio of matched nodes and edges to the total number of those in the ground truth. The following formulas depict how we calculated node and edge accuracy:

$$Node\;Accuracy=\frac{N_{matched}}{N_{ground\;truth}}$$

$$Edge\;Accuracy=\frac{E_{matched}}{E_{ground\;truth}}$$

where $N_{matched}$ refers to the intersection of nodes in the ground truth and those present in the LLM’s output and $N_{ground\;truth}$ refers to the number of nodes in the DOT language files. Conversely, $E_{matched}$ is the number of edges provided by the model’s output that correlates to those present in the ground truth, while $E_{ground\;truth}$ is the number of total edges in the DOT language files.

Node accuracy reflects how well the LLM’s output aligns with the expected nodes, while edge accuracy assesses the correctness of the connections. Finally, we calculated the average accuracy across all compared files to measure overall performance. This method provides a comprehensive evaluation of the LLM’s ability to replicate the detailed structure of the ground truth graphs, ensuring both the correctness of individual components and the overall fidelity of the generated outputs.

## 3. Results

We tested the suitability of our model for retrieving clinical processes based on contextual information with a hypothetical case of a patient presenting with cough and fever, disclosing a recent COVID-19 vaccination. As shown in Table 1, LLM demonstrated promising results by accurately retrieving the diagnosis and suggesting an evaluation for COVID-19 and other relevant respiratory infections. It further advised observation at home unless symptoms necessitated immediate ER evaluation, showcasing its ability to provide nuanced and contextually appropriate recommendations.

In Table 1, we present an example of our results in which our LLM-CDSS suggests the steps that should be taken based on the given context, the patient’s symptoms of cough and fever, and recent COVID-19 vaccination within the past two weeks.

Nineteen additional CPMs were evaluated to measure the model’s token-level accuracy. The average node accuracy across the different pathways was 0.91 (SD ± 0.045), while the average edge accuracy was 0.92 (SD ± 0.122). In Table 2, we present the individual node and edge results per individual CPM.

## 4. Discussion

Compared to conventional approaches, LLM-based retrieval for clinical decision support reveals several advantages. Traditional methods for clinical decisions often involve the manual review of guidelines and consultation with other healthcare professionals, leading to potential delays and inefficiencies in patient care. Some promising alternatives include CDSSs. Current CDSSs assist physicians with diagnosis, disease management, prescription, and drug control [8] and are especially effective in increasing adherence to clinical guidelines and improving patient safety [2,3,7,8]. Their assistance becomes paramount, especially with the rising prevalence of chronic conditions, the emergence of new diseases, and the expansion of medical knowledge, as the demand for healthcare services and documentation increases, resulting in a higher volume of data usage [1,9].

Nevertheless, clinician satisfaction remains low due to excessive time consumption, workflow interruptions, suboptimal EHR integration, irrelevant recommendations, and poor user-friendliness [7,31]. Additionally, studies have demonstrated that physicians perceived that CDSSs increase their cognitive load [7,32] and feel no need for their assistance or disagree with their recommendations [33]. In contrast, LLMs can retrieve relevant information to respond appropriately to user inputs in a natural and fluent human-like conversation [22]. They perform well in information extraction despite not being specifically trained for that task, even outperforming some models in extracting medications and medical evidence [34]. LLMs’ rapid information retrieval and concise recommendations can save valuable clinical time, potentially enhancing overall efficiency and physician acceptance.

These models’ abilities to engage in conversational interactions facilitates their implementation and use in clinical practice as compared to regular AI-based CDSSs [14]. Moreover, while it is a difficult task to ensure that healthcare practitioners read, internalize, and implement new clinical guidelines, the rules implicit in them can be encoded into CDSSs [8] and therefore into LLMs. In this study, we demonstrated the effectiveness of LLMs in retrieving critical information from complex clinical processes, as exemplified by the accuracy of diagnosis and management retrieval in the case of a patient with cough and fever post-COVID-19 vaccination. LLM’s ability to interpret nuanced patient information and provide relevant recommendations showcases its potential utility in clinical decision-making.

Fine-tuned and RAG LLMs have an improved ability to align with the task’s purpose, overcome the base model’s limitations, and be safer, less biased, and harmful. For this reason, they are better suited for application in healthcare and medicine [12,35]. We leveraged a type of RAG technique that allowed us to embed LLaMA 2 with a specialized CPM based on Mayo Clinic’s clinical guidelines and recommendations. RAG enables systems to access external medical databases in real-time to support their knowledge and provide more accurate responses [25]. We used COVID-19 as an example due to its current relevance and rapidly evolving nature, making up-to-date knowledge crucial for precise and safe management. Our model not only accurately retrieved the diagnosis based on the patient’s presentation but also suggested relevant and contextually appropriate evaluations and follow-ups for proper management. Furthermore, it provided a step-by-step explanation of its train of thought, increasing transparency and explainability.

Our results correlate with those of Oniani et al. [36], who also incorporated COVID-19 clinical practice guidelines (CPGs) into different LLMs using three different methods and achieved significant improvements compared to base models. Meanwhile, additional studies have utilized RAG approaches to implement CPGs into different LLMs for nephrology [25], radiology [37], hepatology [38], neurology, gastroenterology, anesthesia and critical care, infectious diseases, and pediatrics [26], all proving to be superior to baseline models for providing accurate, comprehensive, and safe responses. Moreover, some were more time-efficient and less expensive than human personnel [37].

To ensure the precision and reliability of the model’s performance, we compared the token-level accuracy between LLM output and the ground truth. This meticulous comparison allows for a detailed assessment of how accurately the LLM captures and represents specific data elements, particularly critical in high-stakes fields such as healthcare and legal documentation. Token-level accuracy provides insights into the model’s ability to handle nuanced details and identify discrepancies that could have significant implications. By pinpointing and addressing specific areas of deviation, this approach facilitates targeted refinements and enhances the overall quality of the model’s outputs. Furthermore, validating token-level accuracy ensures that the LLM adheres to established standards and benchmarks, reinforcing its applications’ credibility and trustworthiness. Such rigorous evaluation is indispensable for integrating LLMs into critical systems and decision-making processes, where precision and consistency are crucial. With a node and edge accuracy of 0.91 and 0.92, we can determine that the model appropriately understands individual clinical characteristics and their relationships.

LLMs’ potential to become specialized tools, in addition to their natural understanding of quotidian and technical language and embedded logical capacities [11,34,39,40], is particularly important in complex and ambiguous cases where traditional methods might struggle to provide timely and accurate guidance. Our study underscores their potential to augment the clinical decision-making processes such as in managing contagious diseases and other time-sensitive medical conditions. By leveraging LLMs, healthcare providers can streamline information retrieval processes, enhancing patient care and outcomes, as illustrated in Figure 3. Additionally, LLM-based retrieval has the potential to reduce healthcare costs by optimizing resource utilization and minimizing the need for unnecessary diagnostic tests or consultations [13,39,41], ultimately leading to more efficient healthcare delivery and benefiting both patients and healthcare systems [37].

Our results demonstrate the utility of using DOT language for the accurate and efficient retrieval of clinical practice guidelines, in this case, Care Pathway Models. We tested the model on different clinical scenarios, including infectious diseases, chronic diseases, acute and emergent conditions, and medication toxicity across different specialties. This showcases the broad, versatile, and generalizable applicability of LLMs with our proposed framework for clinical decision support across the medical field. Furthermore, this method also opens a broader path as translating clinical or business rules into DOT language establishes a common framework that bridges these rules with LLMs. By implementing this method to embed clinical guidelines or management algorithms into language models, they become capable of being used in a wide array of clinical settings. Depending on the type of clinical process embedded, LLM gains the ability to provide differential diagnoses, treatment options, pertinent laboratory tests and imaging recommendations, and potential prognoses. Given the versatility of DOT language, the models are not limited by specialty or institution, as demonstrated by our results, where the model demonstrated very high accuracy when tested in several clinical settings, from the emergency department to postoperative settings.

Beyond the immediate benefits highlighted in this work, this approach harnesses powerful computing capabilities to work alongside LLMs, enabling the processing of vast and complex rule-based systems in large hospital networks, such as the Mayo Clinic. This integration facilitates a seamless connection between administrative and clinical operations, significantly improving operational costs and, most importantly, freeing physicians from administrative tasks thereby allowing them to devote more time to patient care [12,42].

An additional strength of this approach is that DOT language-encoded business rules are both machine-readable (e.g., by LLMs) and human-readable. This transparency offers substantial advantages in scenarios such as improving and optimizing existing clinical or administrative rules. On top of that, with the assistance of generative AI, this method enables the creation of novel, more efficient rules thereby enhancing the overall rule-authoring process. This dual readability not only ensures accuracy and consistency but also fosters collaboration among stakeholders, enabling the continuous improvement and adaptation of rules to meet evolving clinical and administrative needs. The combination of DOT language and LLMs thus represents a powerful tool in modern healthcare management, dividing innovation and excellence in patient care delivery.

Take, for instance, the creation of new CPMs or updating old ones. At our institution, it is estimated that it may take several months, including gathering subject matter experts (SMEs), building consensus and algorithms, and time for revision. This means that while short CPMs may take up to two weeks, more complex ones can take up to seven months or approximately 100 continuous hours. Conversely, we experimented with the model’s ability to create a new CPM for type 2 diabetes mellitus management based on a straight DOT language structure and obtained an accurate algorithm in just a few minutes. Moreover, when utilizing this new CPM to provide specific patient recommendations for a patient with HbA1c results higher than 7.0%, the LLM retrieved an accurate, straightforward step-by-step guide.

Despite the promising results, there are limitations to consider. It is important to note that the effectiveness of LLMs and therefore LLM-based retrieval is contingent upon high-quality input data and continual model refinement. As these models’ responses are influenced by the data they are trained on, they are subject to reproducing and perpetuating the biases embedded in them [43,44,45]. For instance, if the model is embedded only with CPMs based on a static CPG or on specific patient populations, the model may underperform when implemented in another clinical setting or provide inaccurate advice for patients from minority populations or with extreme clinical conditions [46,47,48,49]. Furthermore, if the models lack contextual understanding, such as models in their basal state, they may generate convincing responses without the proper specificity needed for accurate decision-making, a phenomenon known as hallucination [14]. Although in one study assessing LLMs’ hallucinations, LLaMA and Falcon outperformed other commercial models, such as GTP-3.5, they still hallucinated and produced inaccurate responses, demonstrating their need for further improvement [50].

According to Harrer [51], we must consider six ethical principles when implementing LLMs in clinical practice: accountability, fairness, data privacy and selection, transparency, explainability, and value and purpose alignment. While these models can streamline decision-making, they must not replace the judgment of physicians who will maintain the final responsibility for patient care. Additionally, LLMs must adhere to patient privacy regulations (e.g., HIPAA) to prevent unauthorized access or misuse of sensitive health data. Ensuring robust encryption, strict access controls, and comprehensive audit trails is essential to maintaining patient trust and confidentiality, especially if LLMs will rely on extensive external datasets for their knowledge bases. While this remains a subject of ongoing research, strategies such as prompt engineering, fine-tuning, and RAG may offer a potential solution [25]. Nevertheless, constant model training and validation remain essential to maintaining accuracy and adaptability to evolving medical knowledge and practices.

By providing LLMs with an external curated knowledge database, such as in our methodology and additional RAG approaches, they have access to specialized and up-to-date sources of information that can be tailored to each different clinical setting. This not only improves the models’ accuracy and reduces hallucinations but also increases the interpretability and explainability of the responses, as they are primarily based on information that can be guaranteed to be truthful and accurate [25,52,53,54,55,56]. As shown in Table 1, when a clinician queries the system, the model not only provides a recommendation but also points to a particular section of the CPM. This traceability helps users understand why the model made a certain suggestion. Additionally, this reduces the portion of the model’s logic hidden within its neural algorithm. Moreover, by embedding structured clinical rules and care pathways, the models can present relevant snippets that directly inform the output, allowing decision-makers to see the contextual reasoning steps. However, it is essential to implement human-in-the-loop approaches, where humans constantly evaluate the veracity, safety, and relevance of the information provided to the model and its performance [57].

Finally, it is essential to mention that our results are limited to a few of Mayo Clinic’s Care Pathway Models, limiting their generalizability to other diseases and institutions. Future research in this area could focus on enhancing the interpretability of LLMs in clinical settings. Developing methods to explain the reasoning behind LLM-based recommendations can improve trust and acceptance among healthcare professionals. Exploring new ways to integrate LLMs into existing clinical workflows and decision support systems can further enhance their utility in healthcare settings.

## 5. Recommendations for Healthcare Organizations

For healthcare organizations looking to implement LLM-based information retrieval, several recommendations can be made.

1. Data Quality: Ensure that the input data used to train LLMs is of high-quality and accurately reflects clinical processes and guidelines [12,58].
2. Model Selection: Choose an LLM that is well suited for the specific healthcare application, considering factors such as model performance, scalability, and interpretability [11,16,17,18,40].
3. Integration: Integrate LLMs into existing clinical workflows and decision support systems to maximize their impact and usability [23].
4. Training and Validation: Continuously train and validate LLMs to ensure their accuracy and adaptability to evolving medical knowledge and practices [12,58,59].
5. Collaboration: Collaborate with healthcare professionals and experts to refine LLM-based information retrieval processes and ensure their relevance and effectiveness in clinical settings [12,57,59].

By following these recommendations, healthcare organizations can harness the power of LLMs to enhance information retrieval processes, improve clinical decision-making, and, ultimately, enhance patient care and outcomes.

## 6. Conclusions

This study highlights the potential of LLMs in enhancing information retrieval in clinical processes. The results demonstrate the effectiveness of LLMs in accurately retrieving critical information and providing contextually relevant recommendations, showcasing their utility in clinical decision-making and operational efficiency in healthcare. By addressing limitations and exploring future research directions, LLMs can become valuable and powerful tools in enhancing patient care and optimizing healthcare delivery.

## Figures and Tables

**Table 1:** A sample output of the large language model (LLaMA-2). Please refer to the Supplementary Material for the node names. CPM, Care Pathway Model.

**Query:** A patient, female, 55 years old, visit her physician. Her chief complaints are fever and cough. She said that she had vaccinations two weeks ago. Please give me the next step based on the CPM and the given information.	**Response from LLM:** Based on the context provided, the next steps for a patient with a chief complaint of cough and fever who has had COVID-19 vaccination within the past 14 days would be: Go to node 2 (COVID-19 vaccination in past 72 h?) Since the vaccination was given more than 72 h ago, follow the “NO” path to node 5 (Does patient meet criteria for consideration of COVID-19 or other testing?) Since the patient has symptoms of cough and fever, follow the “YES” path to node 5 (Has patient tested positive for COVID-19 in the past 30 days?) Since we do not have this information, assume “NO” for now and follow the “NO” path to node 16 (Select a pathway based on the presence of fever or symptoms unrelated to a pre-existing condition in the past 48 h) Since the patient has fever, follow the “16 -> 18” path (Unexplained symptoms) Since we do not have enough information to determine if the patient has unexplained symptoms, assume “NO” for now and follow the “NO” path to node 19 (No unexplained symptoms) Since we do not have enough information to determine if the patient has had close contact with a person with confirmed COVID-19 in the past 10 days, assume “NO” for now and follow the “NO” path to node 176 (Has patient tested positive for COVID-19 in the past 30 days?) Since we do not have this information, assume “NO” for now and follow the “NO” path to node 187 (If close contact exposure: Quarantine: Not required Testing: Home antigen testing 5 days after last contact Masking: 10 days after last contact Elective outpatient visits: Allowed) Since we do not have enough information to determine if the patient has had close contact with a person with confirmed COVID-19 or if they have unexplained symptoms or if they have had influenza or group A streptococcal pharyngitis, further evaluation and testing may be necessary based on clinical judgment and the patient’s symptoms and history. Sources: CPM-20522078 (https://askmayoexpert.mayoclinic.org/topic/clinical-answers/prt-20503524/cpm-20522078, accessed on 15 May 2024.)

**Table 2:** The token-level accuracy of LLM outputs compared against the ground truth. The accuracies are measured at the node and edge to delineate the capability of LLM in understanding nodes and relationships (edges).

CPM Name	Node Accuracy	Edge Accuracy
Screening for anal dysplasia and cancer in people living with HIV	0.93	1.00
Nonalcoholic fatty liver disease	0.89	1.00
Mpox	0.94	1.00
Symptomatic severe tricuspid regurgitation: Indications for referral	0.92	0.93
Phenytoin or fosphenytoin order alert logic	0.87	1.00
Pediatric pain management: First-line analgesics, adjunctive therapies, and opioid options	0.90	0.73
COVID-19: Outpatient management (child)	0.91	1.00
Diabetic ketoacidosis or hyperosmolar hyperglycemia state in pregnancy	0.90	1.00
Postpartum hemorrhage (PPH)	0.94	0.71
Differentiated thyroid cancer: Postoperative risk stratification	0.97	0.62
**Myalgic encephalomyelitis/chronic fatigue syndrome (ME/CFS)**	0.88	1.00
Emergency department and inpatient management of atrial fibrillation with rapid ventricular response	0.95	0.79
Management of elevated anion gap acidosis	0.94	0.94
Belzutifan alert logic	0.85	1.00
Sacituzumab-govitecan order alert logic	0.85	1.00
Differentiated thyroid cancer: Radioiodine whole body scan and guide to subsequent management	0.96	0.83
Tricuspid regurgitation	0.97	0.86
COVID-19: Postinfection return to physical activity and sports (child)	0.89	1.00
Preoperative medication management	0.80	1.00
**Average Accuracy**	**0.91**	**0.92**

## Data Availability

The raw data supporting the conclusions of this article will be made available by the authors upon request.

## Supplementary Materials

The following supporting information can be downloaded at: https://www.mdpi.com/article/10.3390/bioengineering12010017/s1, Sample of Care Pathway Model (AskMayoExpert): Code in DOT language.

## Abbreviations

CDSSs	Clinical Decision Support Systems
CPGs	Clinical practice guidelines
CPM	Care Pathway Model
EHRs	Electronic Health Records
JSON	JavaScript Object Notation
LLM	Large language model
RAG	Retrieval-Augmented Generation
SMEs	Subject matter experts
