# Supplementary material for: Using Large Language Models to Retrieve Critical Data from Clinical Processes and Business Rules
Source: Bioengineering (Basel). 2024 Dec 28;12(1):17. doi: 10.3390/bioengineering12010017 (PMC11762383; doi:10.3390/bioengineering12010017)
Supplement: Supplementary file 1 [file bioengineering-12-00017-s001.zip › bioengineering-3353160-supplementary.pdf]

Supplementary Materials

Contents

1. **Sample of Care Pathway Model (AskMayoExpert) code in dot language** 2
2. **References** 4

Sample of Care Pathway Model (AskMayoExpert): code in dot language

```
digraph {
    node[fontsize=16];
    edge[fontsize=16];
    style=filled;
    fillcolor="#D8D8D8";

      // node names
      1 [shape="box" label="1 Outpatient testing"]
      2 [shape="diamond" label="2 COVID-19 vaccination in past 72 hours?"]
      4 [shape="box" label="4 COVID-19 testing recommended only if specific
clinical criteria are present"]
      5 [shape="diamond" label="5 Does patient meet criteria for consideration of
COVID-19 or other testing?"]
      7 [shape="box" label="7 Testing not indicated"]
      16 [shape="box" label="16 Select a pathway based on the presence of fever
or symptoms unrelated to a preexisting condition in the past 48 hours"]
      18 [shape="box" label="18 Unexplained symptoms"]
      19 [shape="box" label="19 No unexplained symptoms"]
      20 [shape="box" label="20 Document whether patient has had close contact
with a person with confirmed COVID-19 in the past 10 days "]
      21 [shape="diamond" label="21 Do symptoms warrant emergency evaluation?"]
      23 [shape="box" label="23 Direct patient to Emergency Department"]
      27 [shape="box" label="27 COVID-19 positive in past 30 days "]
      35 [shape="box" label="35 Offer diagnostic testing for COVID-19 "]
      43 [shape="box" label="43 No diagnosis of COVID-19 in past 30 days "]
      176 [shape="diamond" label="176 Has patient tested positive for COVID-19 in
the past 30 days? "]
      178 [shape="box" label="178 If close contact exposure: Quarantine: Not
required Testing: Not recommended if ≤30 days from prior infection Masking: Not
required beyond the 5 days following isolation discontinuation Elective outpatient
visits: Allowed "]
      187 [shape="box" label="187 If close contact exposure: Quarantine: Not
required Testing: Home antigen testing 5 days after last contact Masking: 10 days
after last contact Elective outpatient visits: Allowed "]
      281 [shape="box" label="281 Inform patient that Mayo Clinic recommends the
use of home antigen tests Proceed with algorithm if patient wishes to be
considered for laboratory-based testing for COVID-19 and other seasonal illness "]
      306 [shape="box" label="306 Select a pathway based on COVID-19 test results
in the past 30 days "]
      309 [shape="box" label="309 COVID-19 testing not indicated "]
      332 [shape="diamond" label="332 Does patient meet criteria to screen for
group A streptococcal pharyngitis testing? "]
      334 [shape="diamond" label="334 Streptococcal pharyngitis screening score
≥3? "]
      337 [shape="diamond" label="337 Does patient meet any criteria for moderate
or high-risk group A streptococcal pharyngitis? "]
      340 [shape="box" label="340 Proceed with previously identified testing
Testing for group A streptococcal pharyngitis not indicated "]
      343 [shape="box" label="343 Add group A streptococcal pharyngitis to
previously identified testing "]
      344 [shape="box" label="344 Assess contraindications for nasopharyngeal
swab, if indicated "]
      345 [shape="box" label="345 If COVID-19 testing is performed, recommend
self-isolation pending test results "]
      360 [shape="box" label="360 Defer all testing Instruct patient to seek
medical care within 24 hours (if moderate risk) or 12 hours (if high risk) "]
      414 [shape="box" label="414 Consider influenza infection "]
```

```
      415 [shape="diamond" label="415 Does patient meet criteria to screen for
influenza testing? "]
      418 [shape="diamond" label="418 Is patient eligible for influenza testing?
"]
      420 [shape="box" label="420 Influenza testing not indicated "]
      422 [shape="box" label="422 Add influenza A/B and RSV to previously
identified testing "]
      423 [shape="box" label="423 Consider group A streptococcal pharyngitis
infection "]


      // connections
      1 -> 2
      2:e -> 4 [label="YES"]
      2:s -> 16[label="NO"]
      4:s -> 5
      5:w -> 16 [label="YES"]
      5:s -> 7 [label="NO"]
      16 -> 18
      16 -> 19
      18 -> 20
      19 -> 176
      20 -> 21
      21:e -> 23 [label="YES"]
      21:s -> 281 [label="NO"]
      27 -> 309
      35 -> 414
      43 -> 35
      176:e -> 178 [label="YES"]
      176:s -> 187 [label="NO"]
      281 -> 306
      306 -> 27
      306 -> 43
      309 -> 414
      332:e -> 340 [label="NO"]
      332:s -> 334 [label="YES"]
      334:e -> 340 [label="NO"]
      334:s -> 337 [label="YES"]
      337:w -> 360 [label="YES"]

      337:s -> 343 [label="NO"]
      340 -> 344
      343 -> 344
      344 -> 345
      414 -> 415
      414:s -> 418 [label="YES"]
      414:e -> 420 [label="NO"]
      418:s -> 422 [label="YES"]
      418:e -> 420 [label="NO"]
      420 -> 423
      422 -> 423
      423 -> 332

}
```

References

1. Mayo Foundation for Medical Education and Research COVID-19 navigator. AskMayoExpert website. (https://askmayoexpert.mayoclinic.org/navigator/covid-19)
2. Parkulo MA, Post JA, Ristagno EH, Tande AJ, Eggers SD, Wald MK. COVID-19 plus seasonal illness: Outpatient testing (adult). (https://askmayoexpert.mayoclinic.org/topic/clinical-answers/prt-20503524/cpm-20522078)
